# The Colombian conflict: a description of a mental health program in the Department of Tolima

**DOI:** 10.1186/1752-1505-3-13

**Published:** 2009-12-23

**Authors:** Elisabeth Sanchez-Padilla, German Casas, Rebecca F Grais, Sarah Hustache, Marie-Rose Moro

**Affiliations:** 1 Epicentre, 8 rue Saint Sabin, 75011, Paris, France; 2 Médecins Sans Frontières France, 8 rue Saint Sabin, Paris, France; 3 Harvard Humanitarian Initiative, Harvard University Cambridge, 14 Story Street, Cambridge, MA 02138, USA; 4 Hôpital Avicenne, Assistance Publique Hôpitaux de Paris, Université de Paris 13, 125 rue de Stalingrad, 93009 Bobigny, France; 5 Hôpital Cochin, Maison des adolescents, Université de Paris 5, 97 Bd de Port Royal, 75679, Paris cedex 14, France

## Abstract

Colombia has been seriously affected by an internal armed conflict for more than 40 years affecting mainly the civilian population, who is forced to displace, suffers kidnapping, extortion, threats and assassinations. Between 2005 and 2008, Médecins Sans Frontières-France provided psychological care and treatment in the region of Tolima, a strategic place in the armed conflict. The mental health program was based on a short-term multi-faceted treatment developed according to the psychological and psychosomatic needs of the population. Here we describe the population attending during 2005-2008, in both urban and rural settings, as well as the psychological treatment provided during this period and its outcomes.

We observed differences between the urban and rural settings in the traumatic events reported, the clinical expression of the disorders, the disorders diagnosed, and their severity. Although the duration of the treatment was limited due to security reasons and access difficulties, patient condition at last visit improved in most of the patients. These descriptive results suggest that further studies should be conducted to examine the role of short-term psychotherapy, adapted specifically to the context, can be a useful tool to provide psychological care to population affected by an armed conflict.

## Findings

Colombia has been seriously affected by an internal armed conflict for more than 40 years. The "guerrillas," the Revolutionary Armed Forces of Colombia (FARC) and the National Liberation Army (ELN), paramilitary groups and the governmental military, control different aspects of the social and political landscape. Civilian populations are the main victims of this conflict, forced to displace, suffering kidnapping, extortion, threats or assassinations.

Due to the security problems, very few medical actors are present and even less mental health professionals are able to work in the region. Médecins Sans Frontières-France (MSFF) has been working in mental health support programs in the department of Tolima, Colombia, since 2002. Tolima, which groups 47 municipalities, is considered a strategic corridor in the armed conflict in Colombia, and has been occupied for more than 30 years.

The MSFF mental health program in Tolima was based on a short-term multi-faceted treatment developed according to the psychological and psychosomatic needs of the population. Here, we describe the population attending during 2005-2008, as well as the psychological treatment provided during this period and its outcomes.

Between 2005 and 2008, MSFF provided psychological care and treatment in Ibague, the capital of the department of Tolima, and in various rural villages. Three mental health teams provided services: two were mobile within the rural areas and one was based in Ibague. They were comprised of expatriate and local psychologists or psychiatrists working jointly with the medical officers.

The population attended differed in these two settings. In Ibague, everyone considered internally displaced by the *Secretaria Departamental de Salud *(regional health authority), was refereed to the MSFF clinic in order to asses their health and psychological condition. Rural villages were included in the program if the civilian population suffered recent or long-term violent events, threats of violent events or if a large number of displaced persons settled there. In Ibague, middle-term psychological treatment with an open schedule of sessions was offered through the mental health ambulatory center. In the rural areas, short-term treatment with a fixed schedule of session (five) was offered through the use of mobile clinics; the first session occurred ideally, less than one month after a violent event. At the last visit, the condition of the patient was assessed and, if considered necessary, they were referred to a governmental institution to continue treatment.

Mental health activities consisted of psycho-informative activities and psychological treatment. The psycho-informative sessions were collective meetings, conducted by a psychologist, where the entire population was invited to attend. These sessions were designed to inform the rural population about the symptoms and consequences of traumatic events, to offer tips and advice on coping mechanisms, and an explanation of the mechanisms of psychotherapy. At the end of the session, the psychologist offered the possibility of an individual consultation. In Ibague, the physician directly referred patients to the psychologist whom they considered candidates for a psychological assessment.

In the individual consultations, patients were asked to complete a practical checklist of symptoms, signs, feelings, as well as to rethink the traumatic events. Patients screening positive for PTSD, anxiety disorders and/or depression, based on criteria from the DSM IV adapted to the Colombian context, were offered individual psychological treatment, and then referred for a more complete psychological assessment. Patients were not included in the psychological program if they presented: chronic psychotic disorders, mental retardation or other mental health conditions not in relationship with the internal armed conflict. In case of exclusion, the patient was referred to the local health system. For those patients admitted, severity of the condition was assessed considering the number and intensity of the signs and symptoms of the disorder, and any resulting impairment in occupational or social functioning. Two types of psychological treatment were offered: individual psychotherapy and therapeutic groups. Individual psychotherapies were based on a short-term psychotherapy model. A rigorous treatment schedule was used in order to assure adherence to the treatment and the fulfillment of the objectives. During each 45-minute session, the psychologist performed therapeutic interventions in order to help the patient understand the relationship between the traumatic events and the current symptoms. Patients were invited to describe their personal history and visual or hearing memories. Group sessions were developed based on a psychotherapy group model. During the first session, patients were informed about the rules of the group therapies, the schedule, the need for confidentiality and the objectives of the treatment. For children, D.W. Winnicott techniques [1,2] were adapted and used; for infants and young children under three, we employed mother-baby dyads. In order to assess difficulties and the functioning of the mother-child interaction [3], we used Lebovici technique based on a psychodynamic brief psychotherapy model [4,5]. In addition to psychotherapy, patients with depression or anxiety that met the following criteria received psychotropic medication: disorders that did not allow the patient to carry out basic daily activities, experience of suicidal ideas, significant disturbances of consciousness, no response or aggravated clinical criteria after psychological treatment. Psychotropic medication was either fluoxetine and/or amytryptiline.

The patient's condition at last visit was classified as aggravated, unchanged, or improved based on the number of DSM IV criteria met and the overall condition reported by the patient and the psychologist. The outcome was defined as improved if a decrease of at least 80% in the total number of DSM IV criteria met initially was observed, or if an improvement regarding daily activities, personal abilities, or capacity for problem-solving compared with the initial evaluation was seen. It was defined as unchanged if the patient did not show changes in the number of DSM IV criteria met or improvement in daily activities, personal abilities, problem solving or coping mechanisms. The clinical outcome was defined as aggravated if there was an increase in the number of criteria met, or there was a new DSM IV diagnoses or if the patients themselves reported aggravated symptoms.

All data were entered into EpiData version 2.0 (EpiData Association, Odense, Denmark). Analysis was conducted using Stata 9.2 (Stata Corporation, College Station, Texas). Medians are given with inter-quartiles range (IQR) [25%-75%] and were compared using the Kruskal-Wallis test. Percentages were compared using the Fisher exact test. Results are presented separately for patients treated in Ibague and in the rural villages.

Between February 2005 and February 2008, the program treated 2,411 people: 855 (35.5%) in Ibague and 1,556 (64.5%) in the villages. The majority of patients were adults (older than 14 years) (75.1%; 1,811/2,411), and women (67.6%; 1,624/2,404). The median (IQR) age for children under 15 was 10 years (8-12), both in Ibague and in the villages. For adults, the median (IQR) age in Ibague and in the rural area was 39 years (28-48) and 40 year (28-52), respectively (Table 1).

**Table 1:** Socio-demographic characteristics of the population treated.

	Ibague (n = 855)		Rural area (n = 1,556)	
	**n**	**%**	**n**	**%**
	
**Sex**				
Male	293	34.4	487	31.4
Female	559	65.6	1,065	68.6
**Age group**				
Under 15	143	16.7	454	29.2
15 or more	713	83.3	1,101	70.8
**Place of residence**				
Ibague	841	98.4	7	0.5
Rural village	14	1.6	1,544	99.5

Department of Tolima, Colombia, 2005-2008.

Among the different traumatic events reported, the most frequent in children in Ibague was being forced to flee, followed by the presence of family violence. In the villages, the most frequent traumatic events reported among children were witnessing murders or physical abuse, having suffered the break up of the nuclear family and suffering family violence. For adults treated in Ibague, having been forced to flee was the main traumatic event reported, followed by having received threats and having lost or destroyed property. In the rural areas, among adults, the most frequent traumatic events were having witnessed murder or physical abuse and having a close family member killed (Table 2).

**Table 2:** Traumatic events reported by the patients.

	Ibague				Rural Areas				p-value
	n		N	%	n		N	%	
Children*									
Sexual violence	11	/	113	9.7	21	/	454	4.6	0.042
Physical injury	4	/	110	3.6	4	/	453	0.9	0.051
Close family member killed	30	/	115	26.1	56	/	454	12.3	0.001
Close family member died from illness	7	/	112	6.3	25	/	454	5.5	0.819
Witness of murder or physical abuse	19	/	123	15.4	85	/	454	18.7	0.431
Received threats	47	/	119	39.5	17	/	454	3.7	<0.001
Incarceration	1	/	111	0.9	8	/	453	1.8	1.000
Property lost or destroyed	45	/	121	37.2	4	/	453	0.9	<0.001
Being forced to flee	137	/	142	96.5	22	/	453	4.9	<0.001
Break-up of nuclear family	51	/	121	42.1	80	/	452	17.7	<0.001
Family violence	110	/	143	76.9	78	/	454	17.2	<0.001
**Adults**									
Sexual violence	47	/	379	12.4	44	/	1,101	4.0	<0.001
Physical injury	32	/	362	8.8	22	/	1,101	2.0	<0.001
Close family member killed	141	/	433	32.6	166	/	1,100	15.1	<0.001
Close family member died from illness	27	/	367	7.4	108	/	1,101	9.8	0.176
Witness of murder or physical abuse	135	/	466	29.0	170	/	1,101	15.4	<0.001
Received threats	590	/	698	84.5	137	/	1,101	12.4	<0.001
Incarceration	4	/	347	1.2	22	/	1,101	2.0	0.362
Property lost or destroyed	432	/	692	62.4	38	/	1,101	3.5	<0.001
Being forced to flee	702	/	710	98.9	80	/	1,101	7.3	<0.001
Break-up of nuclear family	212	/	459	46.2	151	/	1,100	13.7	<0.001
Family violence	135	/	712	19.0	133	/	1,099	12.1	<0.001

Department of Tolima, Colombia, 2005-2008.

* Younger than 15 years old.

The main clinical expression presented was distress or anxiety (39.9%; 943/2,366) and sadness or crying (39.3%; 930/2,366). The most frequent diagnosis was "other anxiety disorder" (32.3%; 750/2,323), which included all anxiety disorders not classified as PTSD or acute stress disorder, followed by depression (18.2%; 423/2,323), acute stress disorder (9.9%; 230/2,323) and PTSD (8.4%; 196/2,323) (Table 3). Most disorders were classified as "moderate", both in children (64.0%; 375/586) and adults (64.3%; 1,144/1,779). The percentage of psychopathologies classified as severe was higher in the villages (14.6%; 220/1,509) than in the city (6.4%; 55/856) (p < 0.001), and in adults (13.0%; 232/1,779) than in children (7.3%; 43/586) (p < 0.001).

**Table 3:** Main clinical expression and diagnosis.

Main clinical expression	Ibague		Rural area		p-value
		
	n	%	N	%	
Children*	**(n = 143)**		**(n = 442)**		
Sadness, crying	33	23.1	90	20.4	0.481
Distress, anxiety	61	42.7	137	31.0	0.011
Inhibition, withdrawal	23	16.1	77	17.4	0.799
Unspecific	22	15.4	127	28.7	0.001
Other	4	2.8	11	2.5	0.768
**Adults**	**(n = 712)**		**(n = 1,069)**		
Sadness, crying	373	52.4	434	40.6	<0.001
Distress, anxiety	247	34.7	498	46.6	<0.001
Inhibition, withdrawal	36	5.1	18	1.7	<0.001
Unspecific	39	5.5	104	9.7	0.001
Other	17	2.4	15	1.4	0.146
**Main diagnosis**					
					
Children*	**(n = 141)**		**(n = 437)**		
Acute stress disorder	24	17.0	6	1.4	<0.001
PTSD	7	5.0	59	13.5	0.006
Other anxiety disorder	16	11.4	159	36.4	<0.001
Depression	20	14.2	21	4.8	0.001
Adjustment disorder	26	18.4	10	2.3	<0.001
Parent-child relational problems	7	5.0	53	12.1	0.016
Other	41	29.1	129	29.5	1.000
**Adults**	**(n = 683)**		**(n = 1,062)**		
Acute stress disorder	173	25.3	27	2.5	<0.001
PTSD	33	4.8	97	9.1	0.001
Other anxiety disorder	94	13.8	481	45.3	<0.001
Depression	139	20.4	243	22.9	0.236
Adjustment disorder	153	22.4	8	0.8	<0.001
Parent-child relational problems	0	0.0	39	3.7	<0.001
Other	91	13.3	167	15.7	0.189

Department of Tolima, Colombia, 2005-2008.

* Younger than 15 years old.

Regarding treatment, both children and adults more frequently received individual psychotherapy (table 4). The median (IQR) number of psychotherapy sessions for children in Ibague was 3 (2-4) and in the villages 2 (1-3). In adults, the median (IQR) number of sessions was 2 (2-4) in Ibague and 2 (1-3) in the villages. In addition to the psychotherapy, 37.0% (407/1,100) and 27.9% (198/711) of adult patients from Ibague and rural areas, respectively, were prescribed psychotropic drugs. This proportion was 11.2% (16/143) and 30.0% (136/454) for children, respectively. Regarding treatment outcome, the most frequent clinical condition in the last consultation was "improved" (table 5).

**Table 4:** Type of psychotherapy received.

	Ibague		Rural area	
**Type of therapy**	**n**	**%**	**n**	**%**

Children*	**(n = 131)**		**(n = 432)**	
Individual	112	85.5	275	63.7
Grouped	7	5.3	111	25.7
Dyad	12	9.2	46	10.7
**Adults**	**(n = 708)**		**(n = 1,099)**	
Individual	691	97.6	976	88.8
Grouped	10	1.4	85	7.7
Dyad	7	1.0	38	3.5

Department of Tolima, Colombia, 2005-2008.

* Younger than 15 years old.

**Table 5:** Clinical condition in the last visit. Department of Tolima, Colombia, 2005-2008

	Ibague		Rural area		p-value
		
Condition in last consultation	n	%	n	%	
Children*	**(n = 117)**		**(n = 324)**		
Aggravated or Unchanged	13	11.11	33	10.19	0.860
Improved	104	88.89	291	89.81	
**Adults**	**(n = 500)**		**(n = 744)**		
Aggravated or Unchanged	47	9.4	61	8.2	0.473
Improved	453	90.6	683	91.8	

* Younger than 15 years old.

There are few studies of mental health in the violent Colombian context [6-8]. Although patients treated in both settings were affected by the same conflict, we observed differences between the urban and rural setting in the traumatic events reported, the clinical expression of the disorders, the most frequent disorders diagnosed, and their severity. These differences may be explained by the displacement itself. Among the displaced living in Ibague, exposure to violent traumatic events was not recent. The urban population faced other challenges related with re-location and urban adaptation. These aspects could explain the higher number of adjustment disorders and acute stress disorders found in the urban area. Similarly, as the conflict was active in the rural area, this may also help explain the higher proportion of severe cases in the rural setting. Examining the correlation between the differences in the experience of traumatic events, symptom severity and diagnoses, although important, is beyond the scope of this study. Our objective was to provide a first descriptive analysis that could provide the basis for future studies.

Although the duration of the treatment was limited due to security reasons and access difficulties, the patient's condition at last visit had improved in most of the patients: over 90% of our patients saw their clinical status improved on their last visit. The absence of a control group did not allow us to develop comparative analysis and therefore we cannot state that this improvement is due solely to the type of psychotherapy received. It would be interesting to look at the role of other factors, such as severity, medication, main diagnosis, although it is beyond the objective of this manuscript to identify predictors of clinical outcome, which might be an interesting second step in the future. A specific data collection and analysis plan would need to be put in place to address these questions.

These descriptive results suggest that further studies should be conducted to examine the role of short-term psychotherapy, adapted specifically to the context, can be a useful tool to provide psychological care to population affected by an armed conflict. Short-term psychotherapy, adapted specifically to the context, through the integration of the culturally variable representations of illness, suffering and treatment [9,10], may be the only viable possibility to offer mental health care in conflict. Other studies should be conducted to elucidate the benefits and constraints of short-term psychotherapy in conflict.

## Ethical considerations

This manuscript is based on routinely-collected data from the MSFF program in Colombia. Authorization for analyzing and publishing the data was sought from the *Secretaría de Salud Departamental de Tolima*.

## Competing interests

The authors declare that they have no competing interests.

## Authors' contributions

ESP had full access to all of the data in the study and takes responsibility for the accuracy of the data analysis. GC and MRM participated in the interpretation of the results. GC, SH, RFG and MRM participated in the critical revision of the manuscript. All authors read and approved the final manuscript.
